# Quercetin Emulsion Ameliorates UVA-Induced Skin via Modulation of NRF2/NF-κB Signaling Pathways

**DOI:** 10.3390/ph19050746

**Published:** 2026-05-09

**Authors:** Jingjing Guo, Zetao Qian, Kai Ji, Hanghang Zhou, Xueyan Wang, Chao Lian, Xiaoqiang Liu, Xuanfen Zhang

**Affiliations:** 1 Department of Plastic Surgery, Lanzhou University, Lanzhou 730000, China; guojj2023@lzu.edu.cn (J.G.); zhouhh2023@lzu.edu.cn (H.Z.); lcwilliam@126.com (C.L.); liuxiaoqiangxq@126.com (X.L.); 2 Department of Orthopedics, Lanzhou University, Lanzhou 730000, China; qianzt2024@lzu.edu.cn (Z.Q.); jik21@lzu.edu.cn (K.J.); 3 Department of Plastic Surgery, The Second Hospital & Clinical Medical School, Lanzhou University, Lanzhou 730030, China; wangxueyan21@lzu.edu.cn

**Keywords:** quercetin, UVA, photoaging, oxidative stress, NRF2, NF-κB, MMPs

## Abstract

**Background:** UVA-induced photoaging is driven by a self-reinforcing cycle of persistent oxidative stress, inflammation, and extracellular matrix (ECM) degradation. Quercetin (Que) offers potent photoprotective potential, yet its clinical utility is hindered by poor aqueous solubility and low skin permeability. **Objective:** To develop a stable quercetin delivery system and evaluate its protective efficacy against UVA-induced photoaging via the NRF2/NF-κB signaling axis. **Methods:** Network pharmacology and molecular docking identified potential targets. An oil-in-water (O/W) nano-emulsion was formulated and characterized. Its effects were evaluated in UVA-irradiated human skin fibroblasts (HSFs; 1.2 J/cm^2^/day for 5 days) and a BALB/c mouse model (20 J/cm^2^/day for 8 weeks). **Results:** Network pharmacology identified 85 shared targets between Quercetin and photoaging. Molecular docking confirmed high affinities (binding energies < −7.0 kcal/mol) for NRF2, NF-κB p65, SOD2, and MMP-1. The optimized O/W nano-emulsion (144–154 nm, Zeta potential −38 to −43 mV) enhanced Quercetin solubility by 175-fold and followed Higuchi release kinetics. In HSFs, 30 μm Quercetin reduced SA-*β*-Gal positivity from 45.8% to 12.5% (73% inhibition), decreased ROS by 66%, and restored Type I collagen intensity to 82 ± 3 a.u. In vivo, topical 0.3% Que emulsion significantly attenuated skin-fold thickening (reducing thickness from 3135 μm to 2170 μm; 30.6% reduction) and achieved a 91% collagen retention rate. Mechanistically, Quercetin treatment significantly upregulated NRF2 and SOD2 expression while suppressing the NF-κB p65/MMP-1/3 inflammatory axis at both mRNA and protein levels (*p* < 0.01). **Conclusions:** Topical Quercetin emulsion effectively facilitates dermal delivery and alleviates UVA-induced photoaging by rebalancing the NRF2/NF-κB axis, thereby enhancing antioxidant defenses and preserving ECM integrity. This formulation represents a robust strategy for skin photoprotection and functional cosmetic intervention.

## 1. Introduction

Skin photoaging is a cumulative degenerative process resulting from prolonged exposure to ultraviolet (UV) radiation. This process significantly compromises cutaneous aesthetics and is intrinsically linked to an elevated risk of diverse photodamage-related dermatological disorders [1,2]. Distinct from intrinsic aging, photoaging is characterized by pronounced deep wrinkling, skin laxity, dyspigmentation, and persistent structural remodeling of the epidermal–dermal junction, often accompanied by the collapse of extracellular matrix (ECM) homeostasis [2,3].

Epidemiological data underscore that UVA (320–400 nm), characterized by its superior dermal penetration and dominance in daily solar irradiance, is the primary driver of most clinical photoaging phenotypes [4]. Unlike UVB, which induces direct DNA lesions, UVA operates predominantly via photochemical mechanisms. It excites endogenous chromophores to generate reactive oxygen species (ROS), which subsequently impair mitochondrial integrity and catalyze the accumulation of oxidative damage [5,6]. Beyond direct biomolecular damage, ROS function as pivotal signaling cues that trigger the sustained activation of stress-responsive pathways. Specifically, the persistent activation of MAPK/AP-1 and NF-κB cascades upregulates pro-inflammatory cytokines and matrix metalloproteinases (MMPs), thereby accelerating collagen degradation and ECM fragmentation [3,7]. Simultaneously, the suppression of the NRF2/ARE-mediated antioxidant defense axis diminishes the expression of cytoprotective enzymes like HO-1, NQO1, and SOD2, thereby impairing the skin’s capacity to neutralize oxidative insults [8].

Crucially, a sophisticated bidirectional crosstalk exists between the NRF2 and NF-κB pathways: NRF2 signaling can antagonize NF-κB-driven inflammatory responses, whereas chronic NF-κB activation may repress NRF2-mediated transcription. Under chronic UVA exposure, this imbalance facilitates a deleterious “oxidative–inflammatory” positive feedback loop [9,10]. Quercetin, a prominent dietary flavonoid, has demonstrated promising photoprotective efficacy owing to its robust antioxidant and anti-inflammatory properties [11,12,13,14]. However, previous investigations have largely focused on its immediate effects on keratinocytes, leaving its impact on long-term UVA-induced dermal structural remodeling and the intricate interplay between NRF2 and NF-κB signaling insufficiently explored [15].

The present study differentiates itself from the existing literature by focusing on dermal fibroblasts—the primary cellular architects of the dermis—within the context of chronic UVA-induced damage. To address the challenge of topical delivery, we utilized a specialized oil-in-water (O/W) emulsion system to enhance the stability and localized delivery of Quercetin. This work systematically examines the concurrent modulation of NRF2 and NF-κB signaling in a unified experimental framework, moving beyond simple correlative observations. By integrating computational network pharmacology and molecular docking with rigorous in vivo and in vitro validation using vehicle-controlled designs, we aim to elucidate the intervention characteristics of this Quercetin-loaded system, thereby providing a more robust mechanistic foundation for its application in dermatological photoprotection.

## 2. Results

### 2.1. Network Pharmacology and Molecular Docking Reveal Potential Key Targets and Pathways

#### 2.1.1. Intersection Targets and PPI Network Analysis

**Figure 1:**
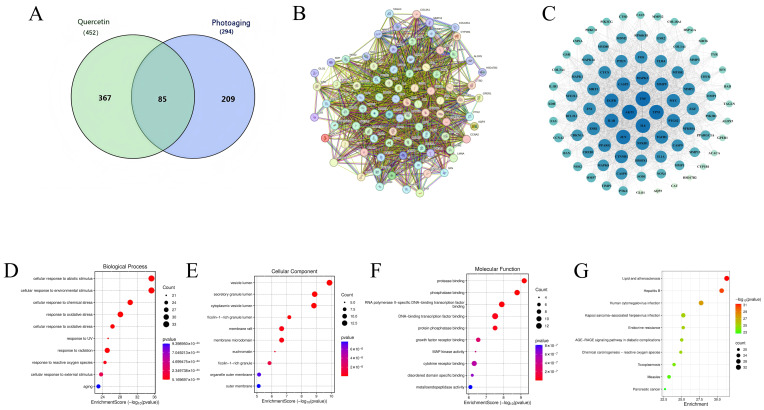
Network pharmacology of quercetin in UVA-induced photoaging. (**A**) Venn diagram illustrating the 85 overlapping targets between quercetin and photoaging-associated genes. (**B**) List of the identified intersection targets. (**C**,**D**) Protein–protein interaction (PPI) network of common targets and its topological analysis; node size and color gradient represent the degree and betweenness centrality, respectively. (**E**,**F**) Gene Ontology (GO) enrichment analysis of overlapping targets across BP, CC, and MF categories. (**G**) KEGG pathway enrichment analysis of overlapping targets.

A total of 452 potential targets for quercetin and 294 photoaging-related genes were identified, with 85 targets found at the intersection (Figure 1A,B). The constructed PPI network comprised 84 nodes and 1661 edges, with topological analysis identifying AKT1, TNF, IL-6, NFKB1, and MMP9 as major hub genes (Figure 1C,D) [16,17]. Based on these topological features and the pathological mechanisms of photoaging, NF-κB, NRF2, SOD2, MMP1, and IL-6 were selected for further experimental validation.

#### 2.1.2. Enrichment Analysis of Oxidative and Inflammatory Pathways

GO enrichment analysis revealed that the 85 intersection targets were significantly involved in the response to oxidative stress, inflammatory response, and collagen catabolic process (Figure 1D–F). KEGG pathway analysis further indicated enrichment in NF-κB, TNF, and PI3K-Akt signaling pathways (Figure 1G). These findings suggest that quercetin intervenes in photoaging by modulating the oxidative stress and inflammation axis.

#### 2.1.3. Molecular Docking Validation of Binding Affinity

Molecular docking confirmed strong binding affinities between quercetin and all five core targets, with binding energies below −7.0 kcal/mol (Figure S1). Quercetin exhibited the highest affinity for NF-κB p65 (−8.2 kcal/mol), forming hydrogen bonds with Arg31, Glu39, and Lys60, alongside π-π stacking with Phe66 (Figure S1). The interaction with NRF2 (−8.0 kcal/mol) involved key residues His333 and Ser412 within the Neh1 domain. Notably, quercetin demonstrated specific coordination with the zinc ion (Zn^2+^) in the catalytic domains of MMP-1 (−7.5 kcal/mol) and IL-6 (−7.8 kcal/mol), potentially inhibiting their enzymatic and pro-inflammatory activities (Figure S1). All docking conformations yielded RMSD values < 2.0 Å, verifying the stability of the predicted binding modes.

### 2.2. Quercetin Emulsion Delivery System Exhibits Nano-Scale Dispersion, Sustained Release, and Short-Term Stability

#### 2.2.1. The Emulsion Forms a Nano-Scale Dispersion System with Relatively Uniform Particle Size Distribution

**Figure 2:**
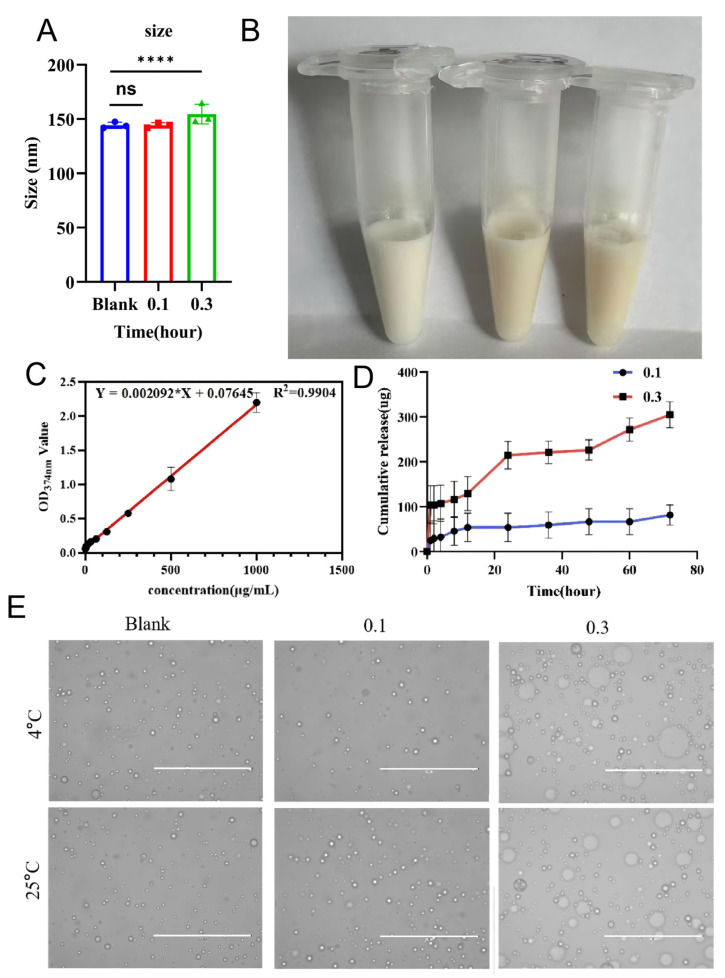
Physicochemical characterization and in vitro release of quercetin emulsions. (**A**) Hydrodynamic particle size and polydispersity index (PDI) of blank emulsion and quercetin emulsions (0.1% and 0.3%, *w*/*w*) measured by dynamic light scattering (DLS). (**B**) Representative photographs of emulsion appearance. (**C**) Quercetin calibration curve determined by HPLC-UV detection at 374 nm. (**D**) In vitro release profiles of 0.1% and 0.3% quercetin emulsions evaluated using a Franz diffusion cell system. (PBS, pH 7.4, 32 °C). (**E**) Representative optical microscopy images (400×) showing emulsion microstructure after short-term storage at 4 °C and 25 °C. Scale bars as indicated. **** *p* < 0.0001; ns, not significant.

Dynamic light scattering (DLS) analysis confirmed that both blank and quercetin-loaded emulsions formed stable nano-dispersed systems. The average droplet sizes for the 0.1% and 0.3% Que groups were 144.25 ± 1.31 nm and 154.53 ± 5.21 nm, respectively (Figure 2A). A polydispersity index (PDI) of approximately 0.2 indicated a narrow and uniform size distribution. Zeta potential values ranged from −38.5 to −42.8 mV (Figure 2A), suggesting robust colloidal stability through electrostatic repulsion. Notably, the emulsion system achieved a 175-fold increase in quercetin solubility (0.35 ± 0.02 mg/mL for the 0.3% group) compared to pure water, with encapsulation efficiencies exceeding 85%.

#### 2.2.2. Standard Curve Shows Good Linearity

The quercetin standard curve, established via HPLC-UV at 374 nm, demonstrated excellent linearity with a regression equation of *y* = 0.002092*x* + 0.07645 and *R*^2^ = 0.9994 (Figure 2C). This validated method provided a reliable basis for quantifying drug content, encapsulation efficiency, and cumulative release in subsequent experiments.

#### 2.2.3. Sustained Release Profiles and Higuchi Kinetic Modeling

In vitro release studies using Franz diffusion cells showed that both quercetin emulsions exhibited sustained release profiles [18]. The cumulative release amount from the 0.3% group was consistently higher than that from the 0.1% group at each time point (Figure 2D). Mathematical modeling demonstrated that the release data adhered strictly to the Higuchi model (*r*^2^ > 0.99), with a release rate constant (k) of 0.031 h^−1/2^ for the 0.3% Que formulation [19]. This confirms that the drug release is primarily governed by a Fickian diffusion mechanism, ensuring prolonged drug availability within the dermal layers.

#### 2.2.4. Quercetin Emulsion Maintains Short-Term Stability at 4 °C and 25 °C

The quercetin emulsion remained stable in appearance and microstructure during storage at both 4 °C and 25 °C. Therefore, the emulsion demonstrates acceptable short-term stability under conventional refrigerated and room-temperature conditions.

### 2.3. Quercetin Treatment Ameliorates UVA-Induced Photoaging Phenotypes and Modulates NRF2/NF-κB Signaling in HSFs

#### 2.3.1. Quercetin Reduces UVA-Induced Cellular Senescence

**Figure 3:**
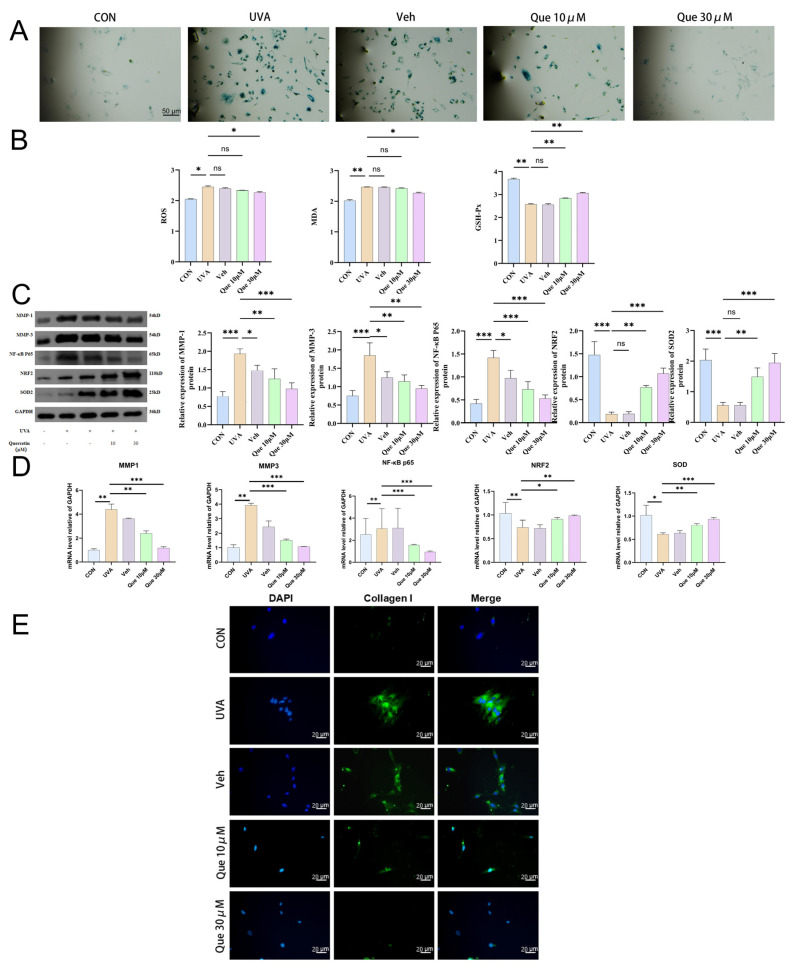
Quercetin mitigates UVA-induced senescence and stress responses in HSFs. (**A**) Representative SA-*β*-Gal staining images (scale bar, 50 μm). (**B**) Quantification of oxidative stress markers (ROS, MDA, and GSH-Px activity). (**C**) Representative immunoblots and densitometric analysis of NRF2, SOD2, NF-κB p65, as indicated), MMP-1, and MMP-3. (**D**) RT-qPCR analysis of mRNA expression of MMP1, MMP3, NF-κB p65, NRF2, and SOD2, normalized to GAPDH. (**E**) Representative immunofluorescence images showing collagen I signals and merged views. Groups: CON, UVA, Veh, Que-10 μM, and Que-30 μM. Data are presented as mean ± SD (*n* = 3 independent experiments). * *p* < 0.05, ** *p* < 0.01, *** *p* < 0.001 vs. UVA; ns, not significant.

UVA irradiation significantly increased the proportion of senescence-associated *β*-galactosidase (SA-*β*-Gal)-positive cells [20,21], from 5.2 ± 0.8% in the CON group to 45.8 ± 3.2% in the UVA group (*p* < 0.01). No significant difference was observed between the UVA + Veh (DMSO) group and the UVA group (*p* > 0.05) (Figure 3A). Compared with the UVA group, treatment with 10μM quercetin significantly reduced the proportion of SA-*β*-Gal-positive cells to 22.1 ± 2.1% (*p* < 0.05), while 30μM quercetin further decreased it to 12.5 ± 1.3% (*p* < 0.01), demonstrating a clear dose-dependent improvement (Figure 3A).

#### 2.3.2. Relief of Oxidative Stress Imbalance: Decreased ROS and MDA, Restored GSH-Px Activity

UVA treatment markedly elevated intracellular ROS and MDA levels while reducing GSH-Px activity; the UVA + Veh group showed no significant difference from the UVA group (*p* > 0.05) (Figure 3B). Compared with the UVA group, 10μM quercetin significantly lowered ROS and MDA levels and increased GSH-Px activity. The 30 μM quercetin group exhibited further enhancement, displaying an obvious dose-dependent trend (Figure 3B).

#### 2.3.3. Upregulation of NRF2/SOD2 Defense and Downregulation of NF-κB/MMP Axis: Consistent Changes at Protein and Transcriptional Levels

UVA significantly downregulated NRF2 and SOD2 protein expression while upregulating phosphorylated NF-κB p65 as well as MMP-1 and MMP-3 (all *p* < 0.01) (Figure 3C). Compared with the UVA group, 10μM quercetin partially restored NRF2/SOD2 levels and reduced MMP-1/MMP-3 expression; 30μM quercetin produced more pronounced improvements (Figure 3C). RT-qPCR results were consistent with the protein changes: compared with the UVA group, both 10μM and 30μM quercetin shifted the mRNA levels of NF-κB p65, MMP1, and MMP3 toward control values, with the 30μM dose showing greater effects (Figure 3D). Overall, quercetin intervention enhanced NRF2/SOD2-related antioxidant defense while suppressing NF-κB/MMP-related readouts (Figure 3C,D).

#### 2.3.4. Quercetin Increases Type I Collagen Expression

UVA irradiation significantly reduced type I collagen fluorescence intensity (Figure 3E). Compared with the UVA group, 10μM quercetin increased collagen I fluorescence intensity, and 30μM quercetin further enhanced it (Figure 3E). These changes were consistent in direction with improvements in oxidative stress markers and NRF2/NF-κB signaling, suggesting that quercetin may help mitigate UVA-induced disruption of matrix homeostasis (Figure 3E).

### 2.4. Topical Quercetin Emulsion Attenuates Chronic UVA-Induced Photoaging Phenotype in Mouse Skin and Regulates NRF2/NF-κB Balance

#### 2.4.1. Quercetin Emulsion Improves Skin Appearance and Suppresses UVA-Induced Skin-Fold Thickening

**Figure 4:**
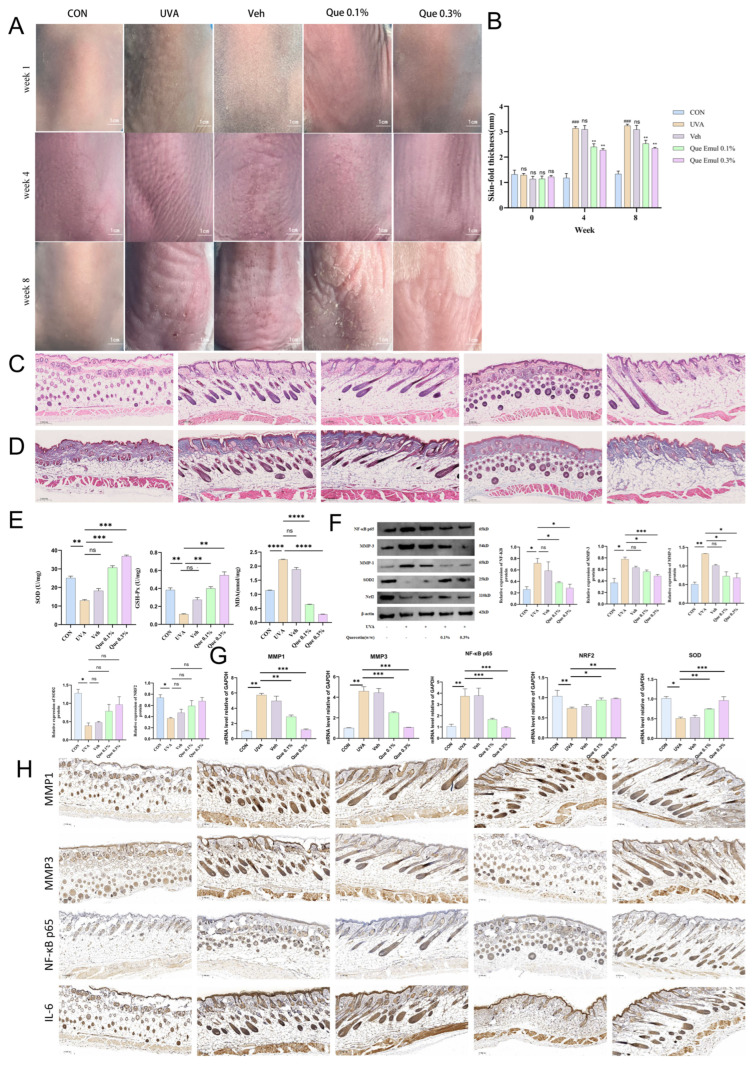
Topical quercetin emulsion attenuates chronic UVA-induced photoaging in BALB/c mouse skin. (**A**) Representative dorsal skin photographs at weeks 1, 4, and 8. (**B**) Longitudinal quantification of skin-fold thickness; the inhibitory effect of 0.3% Que reached 30.6% at week 8. (**C**) Representative H&E-stained skin sections. (**D**) Masson’s trichrome staining showing dermal collagen architecture. (**E**) Oxidative stress markers in skin homogenates (SOD activity, GSH-Px activity, and MDA). (**F**,**G**) Representative immunoblots and RT-qPCR analysis of the NRF2, SOD2, NF-κB p65, MMP-1, and MMP-3 axis. (**H**) Immunohistochemical staining of MMP-1, MMP-3, NF-κB p65, and IL-6 with semi-quantitative analysis (as indicated). Groups: CON, UVA, Veh, Que 0.1%, and Que 0.3%. Data are presented as mean ± SD (*n* = 8 per group). * *p* < 0.05, ** *p* < 0.01, *** *p* < 0.001; **** *p* < 0.0001 vs. UVA; ^###^ *p* < 0.001 vs. CON; ns, not significant.

Chronic UVA irradiation induced pronounced macroscopic skin damage in the UVA group [22], characterized by deep transverse wrinkles, leathery coarseness, and localized erosions. The vehicle group exhibited similar pathological features, confirming the lack of protection from the emulsion base. Topical application of 0.3% Que emulsion significantly mitigated these structural alterations, maintaining a smoother texture with only minimal fine lines (Figure 4A). Longitudinal monitoring of skin-fold thickness revealed a 172% increase relative to baseline in the UVA group by week 8 (rising to 3135 ±12 μm). Quantitative analysis demonstrated that 0.3% Que treatment significantly suppressed this thickening, reducing it to 2170 ± 7 μm (a 30.6% reduction, *p* < 0.01 vs. UVA), with the progression plateauing starting from week 4 (Figure 4B).

#### 2.4.2. Restoration of Histological and Collagen Structural Changes

H&E staining revealed that UVA exposure triggered significant acanthosis and hyperkeratosis, with the epidermal thickness reaching 4.2-fold of the control group (30–50 μm) (Figure 4C). Treatment with 0.3% Que reversed these alterations, reducing epidermal thickness to 1.5-fold of the control (*p* < 0.01) and restoring organized basal cell alignment. Masson’s trichrome staining further demonstrated severe dermal collagen degradation in the UVA group, with the collagen area density (blue pixels) plummeting from 85% (CON) to 48% (*p* < 0.001). In the 0.3% Que group, collagen fibers exhibited enhanced continuity and parallel alignment, with the density successfully restored to 82% (*p* < 0.01), confirming the potent protective effect of the emulsion on dermal matrix integrity (Figure 4D).

#### 2.4.3. Reduction in In Vivo Oxidative Stress Burden: Recovery of SOD and GSH-Px Activities, Decreased MDA

UVA-induced photoaging was accompanied by a systemic failure in redox defense [23], indicated by significantly depleted SOD and GSH-Px activities and an elevation of MDA content to 4.2 ± 0.3 nmol/mg protein (*p* < 0.01) (Figure 4E). Topical intervention with 0.3% Que re-established redox balance, achieving recovery rates of >90% for SOD and GSH-Px activities and a >80% reduction in MDA levels (*p* < 0.01 vs. UVA) (Figure 4E). These findings suggest that Quercetin effectively neutralizes UVA-induced lipid peroxidation within the cutaneous tissue.

#### 2.4.4. Quercetin Emulsion Upregulates NRF2/SOD2 and Downregulates the NF-κB–MMP Axis

Western blot analysis revealed that Quercetin intervention reversed the protein imbalances observed in the UVA group. Specifically, Quercetin upregulated nuclear NRF2 and mitochondrial SOD2 expression (achieving >90% recovery in the 0.3% group) while suppressing the pro-inflammatory and proteolytic signals of *p*-NF-κB p65, MMP-1, and MMP-3 (*p* < 0.01) (Figure 4F). RT-qPCR data were highly consistent with these proteomic findings, showing that the high-dose emulsion repressed the transcription of inflammatory and matrix-degrading genes by 70–80% (Figure 4G). This synergistic modulation of antioxidant and inflammatory pathways corroborates the mechanistic framework established in our in vitro studies.

#### 2.4.5. Immunohistochemistry Indicates Reduced Expression of Inflammation and Matrix Degradation Markers

Immunohistochemical (IHC) staining demonstrated extensive positive signals for MMP-1, MMP-3, NF-κB p65, and IL-6 throughout the epidermis and dermis in the UVA group, particularly in perivascular regions (Figure 4H). Following 0.3% Que treatment, these signals were drastically attenuated, with the integrated optical density (IOD) of positive areas approaching baseline levels, further supporting the efficacy of Quercetin in mitigating UVA-induced matrix disruption.

**Figure 5:**
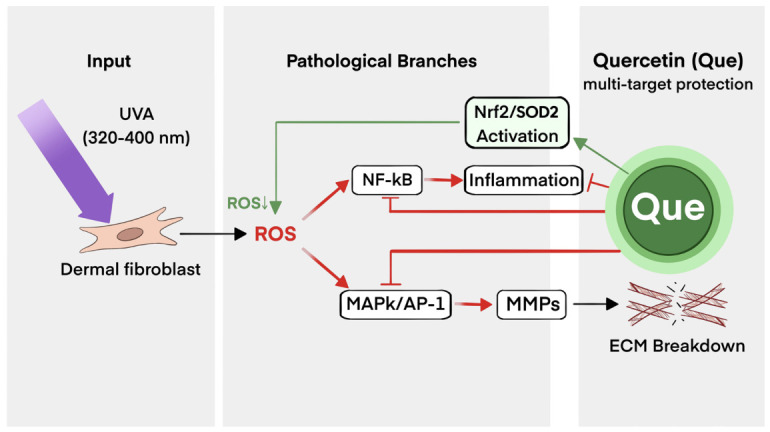
Proposed schematic representation of the multi-target protective mechanisms of Que against UVA-induced skin photoaging. Chronic UVA irradiation (320–400 nm) triggers the excessive production of reactive oxygen species (ROS) in dermal fibroblasts. These ROS subsequently activate diverse pathological cascades, primarily the NF-$\kappa$B-mediated inflammatory response and the MAPK/AP-1/MMPs axis, which collectively drive extracellular matrix (ECM) breakdown and structural remodeling. Topical Que intervention facilitates a synergistic protective effect: (1) it reinforces the antioxidant defense by promoting NRF2/SOD2 activation to neutralize ROS; and (2) it concurrently dampens the pro-inflammatory and proteolytic signals by inhibiting the NF-κB and MAPK/AP-1 pathways. This integrated modulation reestablishes cutaneous redox and inflammatory homeostasis, ultimately alleviating dermal degradation and the photoaging phenotype. Symbols: Green arrows = activation; red arrows = induction of pathological cascades; red T-bars = inhibition; “ROS↓” = reduced ROS levels.

To further integrate the experimental evidence, a comprehensive mechanism schematic was proposed to illustrate how Quercetin coordinates the NRF2NF-κB axis to alleviate UVA-induced photoaging (Figure 5).

## 3. Discussion

Photoaging is characterized by chronic, cumulative skin damage arising from prolonged ultraviolet A (UVA) exposure. Given its deeper dermal penetration and prevalence in solar radiation, UVA plays a critical role in triggering persistent oxidative stress and driving structural remodeling [1,2,24,25]. At the molecular level, chronic UVA exposure is associated with elevated reactive oxygen species (ROS) production, which initiates lipid peroxidation, DNA damage, and the sustained activation of stress-related signaling pathways [5,6,26,27]. These events further upregulate matrix metalloproteinases (MMPs), correlating with the accelerated breakdown of collagen and the extracellular matrix (ECM)—the primary pathological basis for wrinkle formation [7,28]. Furthermore, UVA-mediated impairment of the TGF-*β*/Smad axis suppresses collagen synthesis, shifting matrix homeostasis toward a degradative state [29,30]. Consequently, modulating the key transcription factors governing these processes represents a promising strategy for anti-photoaging intervention [8,9,10,31,32].

In the present study, Quercetin (Que) emulsion treatment exhibited consistent protective effects across both in vitro HSF models and in vivo chronic UVA mouse models. These effects included a reduced oxidative stress burden, enhanced antioxidant defenses, and the suppression of NF-κB-related signaling and its downstream effectors, MMP-1 and MMP-3. These biochemical shifts were accompanied by a marked amelioration of the fibroblast senescence phenotype and skin histological endpoints. Our findings suggest that Quercetin modulates the NRF2/NF-κB balance, thereby mitigating UVA-induced dermal damage.

While previous research has often addressed antioxidant or anti-inflammatory mechanisms in isolation, chronic UVA damage involves a complex, self-reinforcing cycle of both [1,2]. This work demonstrates that Quercetin simultaneously modulates parameters related to both axes, leading to concurrent histological improvements. In our chronic UVA models, we observed the upregulation of NRF2/SOD2 and recovery of GSH-Px activity, concomitant with the downregulation of NF-κB signaling and IL-6 expression. These observations align with prior reports on NRF2-mediated photoprotection in skin [10,31,32] and reinforce Quercetin’s dual-action role within the NRF2/NF-κB axis in chronic exposure scenarios [12,13,14].

Several aspects of this study provide novel contributions to the field. First, unlike many previous studies that prioritized keratinocytes or immune cells [11,14], our research focused on dermal fibroblasts and structural remodeling, directly demonstrating improvements in MMP and collagen-related endpoints—the core pathological hallmarks of photoaging [7,28]. Second, regarding signaling crosstalk, we simultaneously observed NRF2/SOD2 activation and NF-κB suppression within the same experimental system, with consistent trends observed both in vitro and in vivo [7,28]. Third, the delivery strategy addresses a long-standing challenge. Quercetin’s clinical application is often hindered by poor solubility and low skin permeability [13]. By utilizing an optimized O/W nano-emulsion system, we achieved a 175-fold increase in Quercetin solubility, facilitating sustained drug delivery into the dermal layers and enhancing its protective efficacy compared to conventional vehicles [12,33].

The study acknowledges several limitations. First, despite the consistent phenotypic protection observed, direct causal evidence through genetic knockdown or overexpression of NRF2/NF-κB components remains lacking. Thus, the observed effects should be interpreted as modulatory rather than strictly causal at this stage. Second, differences in skin barrier architecture between murine and human models necessitate further validation in ex vivo human skin systems to refine safety margins and translational efficacy [18,19,34]. Third, this study focused on a single compound; exploring synergistic combinations with other polyphenols might further potentiate the observed photoprotective effects [35].

In summary, chronic UVA-induced photoaging is closely linked to a persistent imbalance between oxidative stress and matrix degradation [1,2,3,7,28]. This study provides experimental evidence that topical Quercetin emulsion alleviates UVA-associated dermal remodeling by modulating the NRF2/NF-κB axis, coordinately enhancing antioxidant defenses while suppressing inflammatory responses. These findings support the potential translational application of Quercetin-loaded nano-formulations as effective interventions for skin photoprotection.

## 4. Materials and Methods

### 4.1. Network Pharmacology Analysis

To systematically identify the core nodes through which Quercetin intervenes in skin photoaging, the chemical structure of Quercetin (CID: 5280343) was retrieved from PubChem. Potential targets were predicted via SwissTargetPrediction and STITCH (*Homo sapiens*; similarity threshold > 0.8), followed by standardization using UniProt. Pathological genes associated with skin photoaging were collected from GeneCards, OMIM, and DisGeNET (confidence score > 0.10). The intersection of drug and disease targets was imported into STRING to construct a protein–protein interaction (PPI) network with a confidence score > 0.70. Topological analysis was executed in Cytoscape (version 3.9.1) to identify hub genes based on degree (>10), betweenness centrality, and closeness centrality. Finally, Gene Ontology (GO) and Kyoto Encyclopedia of Genes and Genomes (KEGG) enrichment analyses were performed using DAVID (v6.8) with a significance threshold of *p* < 0.05 to determine the pathways for subsequent experimental validation [16].

### 4.2. Molecular Docking Simulation

Molecular docking was employed to evaluate the binding affinity and interaction modes between Quercetin and identified hub targets. Crystal structures, including NF-κB p65 (PDB ID: 1LE5) and MMP-1 (PDB ID: 1HFC), were obtained from the RCSB Protein Data Bank. Receptor preparation in PyMOL (version 2.5.0) involved removing water molecules and co-crystallized ligands, followed by the addition of polar hydrogens. The Quercetin ligand was subjected to energy minimization via the MMFF94 force field in Chem3D. Docking was executed using AutoDock Vina (version 1.2.0) with a 20 × 20 × 20 Å grid box centered on the validated active sites. The exhaustiveness was set to 8, generating nine poses per run. Beyond the binding energy threshold of <−7.0 kcal/mol, the specificity and stability of the interactions were verified by calculating the root-mean-square deviation (RMSD < 2.0 Å) and analyzing hydrogen bonding, π-π stacking, and zinc ion (Zn^2+^) coordination at the catalytic domains [17].

### 4.3. Preparation and Characterization of Quercetin Emulsion

#### 4.3.1. Emulsion Preparation

Quercetin emulsions (oil-in-water, O/W) were prepared using a two-phase emulsification followed by high-shear homogenization method as previously reported [33]. Emulsions containing 0.1% and 0.3% (*w*/*w*) quercetin were prepared. Quercetin (purity ≥ 98%) was dissolved in a propylene glycol–ethanol mixture with ultrasonication to obtain a clear drug stock solution. The aqueous phase consisted of lecithin, glycerol, and deionized water, premixed thoroughly. The oil phase was composed of liquid paraffin and Tween-80. Both phases were preheated to 50 °C. The oil phase was slowly added dropwise into the aqueous phase under stirring to form a primary emulsion, followed by high-shear homogenization using an IKA T25 homogenizer (10,000 rpm, 2 min) while monitoring temperature to prevent overheating. The quercetin stock solution was then incorporated and mixed uniformly, and the final weight was adjusted to 100.0 g to obtain 0.1% and 0.3% (*w*/*w*) quercetin emulsions. Blank emulsion (without quercetin) was prepared using the same procedure as control. All operations were performed under light protection, and samples were stored at 4 °C.

#### 4.3.2. Appearance and Particle Size Characterization

Emulsion appearance (color, homogeneity, presence of phase separation or precipitation) was visually recorded. Particle size distribution and polydispersity index (PDI), and zeta potential were determined by dynamic light scattering (DLS) [13,33]. Measurements were performed in triplicate, and results are expressed as mean ± standard deviation.

#### 4.3.3. Quercetin Content Determination

Quercetin content in the emulsions was quantified by high-performance liquid chromatography (HPLC). Prior to analysis, emulsion samples were subjected to high-speed centrifugation (4 °C, 12,000 rpm, 30 min) to separate free (unencapsulated) quercetin in the supernatant from encapsulated quercetin in the precipitate. Both fractions were dissolved in methanol with ultrasonication, filtered (0.22 μm), and analyzed.

Chromatographic conditions: Agilent 1260 system; C18 column (4.6 × 250 mm, 5.0 μm); mobile phase methanol/water (70:30, *v*/*v*); flow rate 1.0 mL/min; detection wavelength 374 nm.

A standard curve was established using quercetin reference standard at various concentrations, with peak area plotted against concentration (linear regression, R^2^ ≥ 0.99). Total drug content was determined after complete demulsification with methanol (vortexing and ultrasonication to fully dissolve quercetin), followed by HPLC analysis [13,33]. Free drug content was quantified from the supernatant after centrifugation. Encapsulation efficiency (EE%) was calculated as:

*EE*% = [(*W*_total_ − *W*_free_)/*W*_total_] × 100%.

#### 4.3.4. In Vitro Transdermal Permeation Study (Franz Diffusion Cell) 

In vitro skin permeation of quercetin emulsions was evaluated using Franz diffusion cells [18,19]. The receptor compartment was filled with pH 7.4 PBS maintained at 37 °C with magnetic stirring (300 rpm). The effective diffusion area was 2.27 cm^2^. Full-thickness dorsal skin from BALB/c mice was used after hair removal, subcutaneous fat and connective tissue excision, PBS rinsing, and surface drying. Skin was mounted between donor and receptor compartments with the stratum corneum facing the donor side and dermis facing the receptor. Air bubbles were carefully removed. The receptor compartment was filled with 15.0 mL PBS. One milliliter of sample (blank emulsion, 0.1%, or 0.3% quercetin emulsion) was applied to the donor compartment. Samples (2.0 mL) were withdrawn from the receptor at predetermined time points (5, 10, 20, 30 min, and 1, 2, 3, 12, 24, 36, 48, 60, 72, 84 h) and replaced with an equal volume of fresh PBS to maintain constant volume. Receptor samples were analyzed by HPLC-UV at 374 nm, and cumulative permeation amounts were calculated based on the standard curve. Permeation profiles were plotted accordingly. To determine the release mechanism, cumulative data were fitted to the Higuchi mathematical model (*Q* = *kt*^1/2^), and the coefficient of determination (r^2^) was calculated to assess the goodness of fit.

#### 4.3.5. Stability Evaluation

Stability was assessed under storage at 4 °C and 25 °C for 4 weeks. Appearance, pH, particle size, and quercetin content were monitored at weeks 0, 1, 2, and 4 [12,13].

### 4.4. Cell Culture

Human dermal fibroblasts (HDFs, ATCC, CRL-2097) were purchased from the American Type Culture Collection (ATCC, Manassas, VA, USA) as primary cells derived from adult foreskin tissue. Cells were cultured in Dulbecco’s Modified Eagle Medium (DMEM; Gibco, Grand Island, NY, USA) supplemented with 15% fetal bovine serum (FBS; BI), 100 U/mL penicillin, and 100 μg/mL streptomycin. Cultures were maintained at 37 °C in a humidified incubator with 5% CO_2_ (Thermo Fisher Scientific, Waltham, MA, USA).

### 4.5. Chronic UVA Irradiation Model

The chronic UVA irradiation model was established following the method described by Montoni [20]. Cells were seeded into culture plates and grown to approximately 70% confluence before irradiation. The control group was covered with aluminum foil to block light exposure. For UVA irradiation, the culture medium was removed, cells were gently washed with PBS, and sufficient PBS was added to cover the cell surface to prevent drying during irradiation. Irradiation was performed in a biosafety cabinet with the plate lid removed, using a UVA lamp source (Guanya, Shenzhen, China). The irradiation intensity was 0.62 mW/cm^2^, with the light source positioned approximately 30.0 cm from the plate. Each irradiation session lasted 32.0 min (delivering 1.2 J/cm^2^ per day). Irradiation was repeated once every 24 h for 5 consecutive days, resulting in a cumulative dose of 6.0 J/cm^2^. During irradiation, airflow was maintained to assist heat dissipation and minimize temperature rise. Immediately after each irradiation, PBS was discarded and replaced with fresh complete culture medium. Samples were collected 24 h after the final irradiation for subsequent analyses.

### 4.6. Grouping and Treatment Protocol

Cells were divided into the following groups: control (CON), vehicle (Veh; <0.1% DMSO), UVA model (UVA), low-dose quercetin (Que 10 μM), and high-dose quercetin (Que 30 μM). The CON group received no UVA irradiation. The UVA group received UVA irradiation only. Quercetin was prepared as a stock solution in DMSO and diluted in complete medium to working concentrations of 10.0 μM and 30.0 μM immediately before use. Following each UVA irradiation, PBS was removed and replaced with fresh complete medium containing quercetin (final concentrations 10.0 μM or 30.0 μM). Cells were then incubated for an additional 24 h before detection. The DMSO concentration was maintained below 0.1% (v/v) across all groups to eliminate solvent interference [11,14].

### 4.7. Cellular Senescence Detection: SA-β-Gal Staining

Cellular senescence was assessed using a senescence-associated *β*-galactosidase (SA-*β*-Gal) staining kit (KTA3030; Abbkine, Wuhan, China) according to the manufacturer’s instructions and established protocols [21]. Cells were fixed with fixation solution at room temperature for 15 min, washed with PBS, and incubated with SA-*β*-Gal working solution overnight at 37 °C in a CO_2_-free incubator under light protection. The following day, cells were washed with PBS and observed under an inverted microscope (Olympus, Tokyo, Japan). Images were captured at 400× magnification, and SA-β-Gal-positive cells were quantified in 10 randomly selected fields per sample. Image acquisition and counting were performed in a blinded manner using coded files.

### 4.8. Oxidative Stress Detection: ROS, GSH-Px, and MDA

Intracellular reactive oxygen species (ROS) levels were measured using the DCFH-DA probe method. After treatment, culture medium was discarded, cells were washed twice with PBS, and DCFH-DA probe (S0033; Beyotime, Shanghai, China) was added and incubated at 37 °C in the dark for 20 min. Cells were then washed with PBS and imaged under an inverted fluorescence microscope. Lipid peroxidation was quantified by measuring malondialdehyde (MDA) levels using an MDA assay kit (ab118970; Abcam, Cambridge, UK). Glutathione peroxidase (GSH-Px) activity was determined using a GSH-Px assay kit (ab205887; Abcam, Cambridge, UK). All assays were performed according to the manufacturers’ instructions [26,27]. Results were normalized to total protein content (determined by BCA method) and expressed relative to the control group.

### 4.9. Protein Expression Detection: Western Blot

Total cellular protein was extracted using RIPA lysis buffer (P0013B; Beyotime, Haimen, Jiangsu, China) supplemented with protease inhibitors (P1005) and phosphatase inhibitors (P1081). Cells were lysed on ice, followed by centrifugation at 4 °C to collect the supernatant. Protein concentration was determined by the BCA method (PC0020; Solarbio, Beijing, China). Equal amounts of protein were separated by SDS-PAGE and transferred to membranes. After blocking, membranes were incubated with primary antibodies (see Table S1 for details; antibodies were purchased from Abcam, Proteintech, or CST and used at standardized working dilutions). Subsequently, HRP-conjugated secondary antibodies were applied, followed by chemiluminescent detection. Band images were captured, and grayscale values were quantified using ImageJ software (version 1.53; NIH, Bethesda, MD, USA) [29]. Target protein signals were normalized to GAPDH.

### 4.10. Gene Expression Detection: RT-qPCR

Total RNA was extracted using an RNA extraction kit (R6834-01; Omega Bio-Tek, Kusatsu, Japan). Reverse transcription was performed to synthesize cDNA, followed by real-time quantitative PCR (qPCR) amplification. mRNA expression levels of NRF2, SOD2, NF-κB p65, MMP1, and MMP3 were determined, with GAPDH serving as the internal reference. Relative expression was calculated using the 2^−ΔΔCt^ method [29,30]. Primer sequences are provided in Table S2.

### 4.11. Immunofluorescence and Confocal Imaging: Type I Collagen

Cells were fixed with 4% paraformaldehyde, permeabilized, and blocked. They were then incubated with anti-Type I collagen primary antibody (1:200, Abcam), followed by Alexa Fluor 488-conjugated secondary antibody (1:500, Invitrogen, Carlsbad, CA, USA). Nuclei were counterstained with DAPI [29,30]. Images were acquired using a laser scanning confocal microscope (Olympus FV3000; Olympus Corporation, Tokyo, Japan) and analyzed for mean fluorescence intensity.

### 4.12. Animal Experiments

#### 4.12.1. Animals and Ethics

Female BALB/c mice (20–23 g, 6–8 weeks old, specific pathogen-free grade) were purchased from the Laboratory Animal Center of Lanzhou University (Lanzhou, China) and housed under SPF conditions with free access to food and water. All animal experimental procedures were approved by the Animal Ethics Committee of Lanzhou University (approval number: D2025-530).

#### 4.12.2. Grouping and Time Points

Mice were randomly divided into the following groups (*n* = 8 per group): control (CON), UVA model (UVA), vehicle emulsion control (Veh; blank emulsion without quercetin), 0.1% quercetin emulsion (Que Emul 0.1%), and 0.3% quercetin emulsion (Que Emul 0.3%). The CON group received no UVA irradiation and no topical treatment. The UVA group received only UVA irradiation. The Veh group received UVA irradiation and topical application of blank emulsion (identical vehicle formulation without quercetin). The Que Emul 0.1% and Que Emul 0.3% groups received UVA irradiation plus topical application of 0.1% (*w*/*w*) or 0.3% (*w*/*w*) quercetin emulsion, respectively. All animals were maintained under identical housing conditions and received the corresponding treatments. The experimental duration was 8 weeks. Skin phenotype (appearance and fold thickness) was evaluated at weeks 1, 4, and 8. At the end of week 8, terminal assessments were performed, after which mice were euthanized for tissue collection and subsequent histological and molecular analyses.

#### 4.12.3. Chronic UVA Irradiation Protocol

Prior to irradiation, the dorsal hair of mice was removed by shaving, and the irradiation area was cleaned. Mice were then exposed to UVA (315–400 nm) using a UVA lamp (Guanya, Shenzhen, China) at an irradiance of 1.3 mW/cm^2^ for 4.3 h per day (approximately 20.0 J/cm^2^ per day), with the light source positioned about 30.0 cm from the skin [22]. A restraint device was used to maintain consistent exposure of the dorsal skin without anesthesia. The control group underwent the same shaving and restraint procedures but was not irradiated. Irradiance output was calibrated before each session using a radiometer, and stability was monitored during irradiation [22]. Body weight was recorded before and after each irradiation to monitor general condition and detect potential adverse effects. Irradiation was performed 5 days per week for 8 consecutive weeks. All quantitative assessments were conducted in a blinded manner by an independent investigator.

#### 4.12.4. Quercetin Emulsion Intervention

Five minutes after each UVA irradiation, the corresponding emulsion was evenly applied to the irradiated dorsal area at a dose of 0.1 mL/cm^2^. The control and UVA groups received no topical application, but all other handling procedures were identical.

#### 4.12.5. Appearance and Skin-Fold Thickness Measurement

Representative photographs of the dorsal skin were taken at weeks 1, 4, and 8. Skin-fold thickness was determined using a digital caliper by gently lifting a double-layer skin fold along the midline [23]. Measurements were repeated three times per mouse and averaged.

#### 4.12.6. Histology and Immunohistochemistry (IHC) 

At the end of week 8, mice were euthanized by intraperitoneal overdose of sodium pentobarbital. Full-thickness dorsal skin from the irradiated area was collected after removal of subcutaneous fat and fascia, and divided for different analyses. Portions were fixed in 4% paraformaldehyde for >24 h, dehydrated, cleared, paraffin-embedded, and sectioned at 4 μm thickness for H&E and Masson’s trichrome staining to evaluate epidermal thickness, collagen fiber arrangement, and dermal structural changes. For immunohistochemistry, sections were deparaffinized, rehydrated, subjected to antigen retrieval (citrate buffer pH 6.0 per antibody instructions), blocked with 3% H_2_O_2_ to inhibit endogenous peroxidase, and incubated with primary antibodies (details in Figure S1; antibodies from Abcam, Proteintech, or Bioss at standardized dilutions). HRP-conjugated secondary antibodies were applied, followed by DAB chromogenic development and hematoxylin counterstaining. Images were captured under uniform microscope settings. At least five random fields per section were quantified at the same magnification using consistent threshold and ROI criteria.

#### 4.12.7. Oxidative Stress Detection in Tissue: SOD, GSH-Px, and MDA

Full-thickness dorsal skin from the irradiated area (subcutaneous fat and fascia removed) was processed on ice. Tissue was homogenized in pre-chilled PBS or kit-specific homogenization buffer and centrifuged to collect supernatant. SOD activity (ab65354; Abcam, Cambridge, UK), GSH-Px activity (ab102530; Abcam, Cambridge, UK), and MDA content (ab118970; Abcam, Cambridge, UK) were measured using commercial kits (Abcam, Cambridge, UK) according to the manufacturers’ instructions. Results were normalized to total protein content of the corresponding samples.

#### 4.12.8. Tissue Protein Detection: Western Blot

Total protein from dorsal skin tissue (subcutaneous fat and fascia removed) was extracted using RIPA lysis buffer (P0013B; Beyotime), quantified by BCA method (PC0020; Solarbio), and subjected to Western blot analysis (primary antibodies as listed in Table S1). Equal amounts of protein were separated by SDS-PAGE, transferred to membranes, blocked, and sequentially incubated with primary and HRP-conjugated secondary antibodies. Chemiluminescence was used for detection, and band grayscale values were quantified using ImageJ with normalization to GAPDH.

#### 4.12.9. Gene Expression Detection: RT-qPCR

Total RNA was extracted from tissue, reverse-transcribed to cDNA, and subjected to real-time qPCR. mRNA expression levels of NRF2, SOD2, NF-κB p65, MMP1, and MMP3 were measured with GAPDH as the internal reference. Relative expression was calculated using the 2^−ΔΔCt^ method (primer sequences as in Table S2).

### 4.13. Statistical Analysis

Statistical analysis and graphing were performed using GraphPad Prism 9.0 and SPSS 26.0 software. Data are presented as mean ± standard deviation (mean ± SD). For multiple comparisons, one-way ANOVA followed by Bonferroni post hoc tests was employed. Longitudinal data (weeks 1, 4, and 8) were analyzed using repeated-measures ANOVA with Bonferroni correction. *p* < 0.05 was considered statistically significant.

## 5. Conclusions

This study demonstrates that an optimized O/W nano-emulsion significantly enhances the solubility (175-fold) and dermal delivery of Quercetin, effectively overcoming its primary translational limitation. Our findings confirm that topical intervention with this system ameliorates chronic UVA-induced skin photoaging by orchestrating the NRF2/NF-κB signaling axis. By concurrently boosting NRF2-mediated antioxidant defenses and suppressing NF-κB-driven matrix degradation, this formulation restores collagen integrity and preserves dermal architecture. These results establish the Quercetin-loaded nano-emulsion as a robust and high-performance candidate for dermatological photoprotection, providing a mechanistic framework for the clinical development of flavonoid-based anti-aging interventions.

## Data Availability

The data presented in this study are available on request from the corresponding author. The data are not publicly available due to privacy and ethical restrictions.

## Supplementary Materials

The following supporting information can be downloaded at: https://www.mdpi.com/article/10.3390/ph19050746/s1, Table S1: Primary antibodies used for Western blot; Table S2: Primer sequences used in real-time PCR; Figure S1: molecular docking identify candidate targets of quercetin in UVA-induced photoaging.
