# Supplementary material for: Quercetin Emulsion Ameliorates UVA-Induced Skin via Modulation of NRF2/NF-κB Signaling Pathways
Source: Pharmaceuticals (Basel). 2026 May 9;19(5):746. doi: 10.3390/ph19050746 (PMC13210000; doi:10.3390/ph19050746)
Supplement: Supplementary file 1 [file pharmaceuticals-19-00746-s001.zip › pharmaceuticals-4228329-supplementary.pdf]

Table S1. Primary antibodies used for Western blot

Target	Supplier	Catalog No.	Dilution
NRF2	Abcam	ab62352	1:1000
SOD2	Proteintech	24127-1-AP	1:2000
p-NF-κB p65	Cell Signaling Technology	8242	1:1000
MMP-1	Abcam	ab134184	1:1000
MMP-3	Abcam	ab52915	1:1000
GAPDH	Proteintech	60004-1-Ig	1:5000

Table S2. Primer sequences used in real-time PCR

Gene	Primer（5′→3′）
**NRF2**	**F:** AGTGGCAGGAGGCAGTATGA<br>**R:** TGCTCAATGTCCTGTTGCAT
**SOD2**	**F:** GCTGGAGAAGGTGGAAAGGA<br>**R:** GCAATGTGAGCTTCCCAGTT
**NF-κB p65**	**F:** GCCAGGAGACTTTCCGATGT<br>**R:** GGTCCCGTGAACTTCACCAT
**MMP-1**	**F:** CCTGGATACCAAGATGTTCCC<br>**R:** TGGCATTGAGCCTTGGTACT
**MMP-3**	**F:** CAGCTACTTTCCCAGCAAGA<br>**R:** CTTCATGCGTAGGAGGTCCA
**GAPDH**	**F:** TGTGGGCATCAATGGATTTTG<br>**R:** ACCCCAATACGACTCAATCC

Figure S1. molecular docking identify candidate targets of quercetin in UVA-induced photoaging.

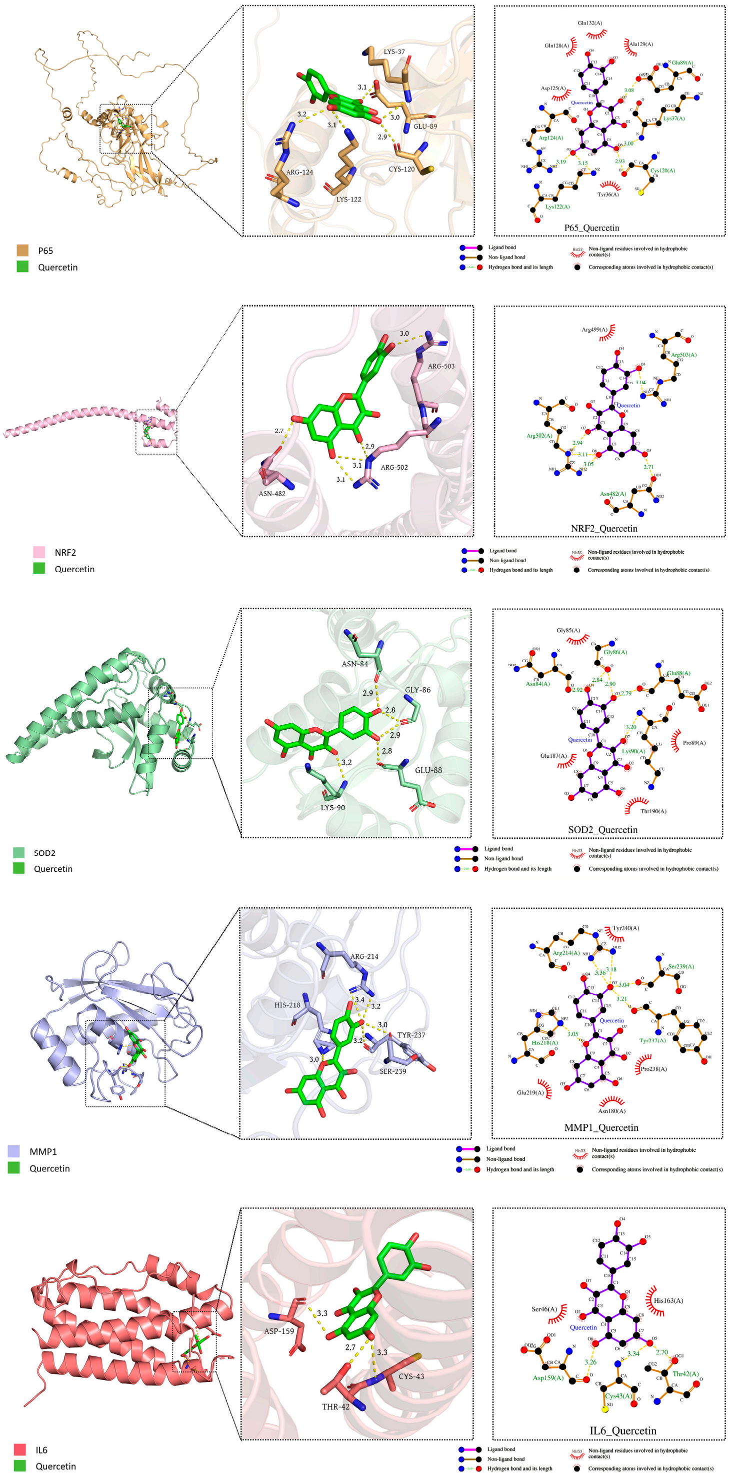

Molecular docking models showing the stable binding conformations of quercetin with core targets: NF-κB p65, NRF2, SOD2, MMP1, and IL-6. Key interactions, including hydrogen bonds, π-π stacking, and zinc ion coordination, are highlighted.
